# Detection and Localization of IL-8 and CXCR1 in Rainbow Trout Larvae in Response to *Pseudomonas aeruginosa* Lipopolysaccharide

**DOI:** 10.3390/ani14192878

**Published:** 2024-10-06

**Authors:** Paula A. Santana, Juan C. Forero, Fanny Guzmán, Sandra Gaete, Félix Acosta, Luis A. Mercado, Claudio A. Álvarez

**Affiliations:** 1 Instituto de Ciencias Aplicadas, Facultad de Ingeniería, Universidad Autónoma de Chile, Santiago 8910060, Chile; paula.santana@uautonoma.cl; 2 Laboratorio de Bioingeniería de Tejidos e Innovación Odontológica, Facultad de Odontología, Universidad de Valparaíso, Valparaíso 2360004, Chile; juancarlos.forero@uv.cl; 3 Cátedra de Ciencias Básicas, Escuela de Odontología, Facultad de Odontología, Universidad de Valparaíso, Valparaíso 2360004, Chile; 4 Núcleo Biotecnología Curauma, Pontificia Universidad Católica de Valparaíso, Valparaíso 2340025, Chile; fanny.guzman@pucv.cl; 5 Laboratorio de Diagnóstico de COVID-19, Unidad de Detección y Análisis, Universidad de O’Higgins, Rancagua 2841959, Chile; sandra.gaete.g@gmail.com; 6 Grupo de Investigación en Acuicultura (GIA), Instituto Universitario Ecoaqua, Universidad de Las Palmas de Gran Canaria, Islas Canarias, 35214 Taliarte, Spain; felix.acosta@ulpgc.es; 7 Instituto de Biología, Pontificia Universidad Católica de Valparaíso, Valparaíso 2340025, Chile; 8 Laboratorio de Cultivo de Peces, Departamento de Acuicultura, Universidad Católica del Norte, Coquimbo 1781421, Chile; 9 Laboratorio de Fisiología y Genética Marina (FIGEMA), Centro de Estudios Avanzados en Zonas Áridas (CEAZA), Coquimbo 1781421, Chile

**Keywords:** trout larvae, IL-8, CXCR1, LPS, immunological capacity

## Abstract

**Simple Summary:**

The salmonid industry is susceptible to opportunistic pathogens particularly affecting fish in early developmental stages. Understanding the immunological capacity during these stages is crucial for effective disease control. The receptor for Interleukin 8 (CXCR1), plays a key role in various inflammatory conditions. This study analyzed the expression of IL-8R and IL8 in rainbow trout larvae in response to bacterial lipopolysaccharide (LPS). The larvae 19 day post-hatching (dph) exhibited strong immune responses after 8 h of LPS stimulation, with IL-8 and omCXCR1 protein expression increasing. The use of specific antisera allowed localization of both proteins in mucosal tissue cells. This study highlights the effectiveness of LPS immersion in activating the innate immune system of trout larvae using IL-8/CXCR1 as immunological markers.

**Abstract:**

The salmonid industry faces challenges due to the susceptibility of fish to opportunistic pathogens, particularly in early developmental stages. Understanding the immunological capacity during these stages is crucial for developing effective disease control strategies. IL-8R, a member of the G-protein-coupled receptor family, acts as a receptor for Interleukin 8 (IL-8). The binding of IL-8 to IL-8R plays a major role in the pathophysiology of a wide spectrum of inflammatory conditions. This study focused on the immune response capacity of rainbow trout (*Oncorhynchus mykiss*) larvae by analyzing IL-8/CXCR1 response to lipopolysaccharide (LPS) from *Pseudomonas aeruginosa*. Previous research demonstrated that LPS from *P. aeruginosa* acts as a potent immunostimulant in teleost, enhancing pro-inflammatory cytokines. The methodology included in silico analysis and the synthesis and characterization of an omCXCR1-derived epitope peptide, which was used to produce omCXCR1-specific anti98 serum in mice. The research revealed that rainbow trout larvae 19 days post-hatching (dph) exhibited pronounced immune responses post-stimulation with 1 µg/mL of LPS. This was evidenced by the upregulated protein expression of IL-8 and omCXCR1 in trout larvae 2 and 8 h after LPS challenge, as analyzed by ELISA and immunohistochemistry. Furthermore, fluorescence microscopy successfully revealed the colocalization of IL-8 and its receptor in cells from mucosal tissues after LPS challenge in larvae 19 dph. These findings underscore the efficacy of LPS immersion as a method to activate the innate immune system in trout larvae. Furthermore, we propose IL-8 and its receptor as molecular markers for evaluating immunostimulation in the early developmental stages of salmonids.

## 1. Introduction

The cultivation and production of rainbow trout (*Oncorhynchus mykiss*) have increased its demand for consumption worldwide, representing one of the main exports in aquaculture for countries like Chile and Norway [1]. However, the recurrent presence of opportunistic pathogens causes important economic losses in the salmon industry, particularly in the early developmental stages of the fish, where the highest mortality rates are ascribed to a higher susceptibility to infections [2].

Successful larval cultivation ensures sustainable large-scale production. Thus, to evaluate new strategies to increase survival in freshwater larval cultures of salmonids, a proper understanding of the immunological capacity of fish during these developmental stages is required. It has been described that during their early stages of development fish rely mainly on passive immunity of maternal antibodies, together with innate immunity, as the main defence mechanisms against pathogenic microorganisms [3,4,5,6,7]. Studies conducted in young post-hatch rainbow trout larvae demonstrated their ability to respond immunologically to an infection with *Ichthyophthirius multifiliis*, being able to regulate immune-relevant genes, like cytokines, chemokines, and acute phase proteins [8]. Additionally, the capacity of immune response in both 17- and 87-day post-hatch (dph) larvae and fry in response to *Yersinia ruckeri* was analysed. It was observed that in 17 dph larvae few genes are expressed, revealing a limited capacity of the larvae; however, in 87 dph fry, high gene expression of cytokines, acute phase proteins, complement factors, antimicrobial peptides (AMPs) and inducible nitric oxide synthase (iNOS) was observed in response to *Y. ruckeri* [9].

The genus *Pseudomonas* is one of the most diverse and ecologically significant bacterial groups, known for its remarkable ability to thrive in a wide range of habitats, including freshwater environments [10]. These microorganisms can also grow well at low temperatures, which contributes to their frequent presence in the normal microbiota of salmonids [11]. However, under stress conditions such as malnutrition and overcrowding, *Pseudomonas* can be highly opportunistic and pathogenic, leading to severe diseases, like haemorrhagic septicaemia and gill necrosis [12]. Due to their pathogenic potential, many studies have used the lipopolysaccharides (LPSs) of *Pseudomonas* as a model to investigate the immune response of fish against these opportunistic pathogens. In this regard, our research group studied the immune response capacity of rainbow trout larvae at 33, 42, 56, and 66 dph, where, at the transcript and protein level, larvae expressed at certain times AMPs, such as hepcidin and cathelicidin, in addition to the chemokine IL-8 against LPS of *Pseudomonas aeruginosa*. This demonstrates that fish larvae possess an immune response capacity, although this is dependent on the stage of trout development [13]. In the above mentioned studies, IL-8 or CXCL8_L1 [14] are cytokines of great relevance, being able to rapidly increase their expression levels in response to pathogen-associated molecular patterns (PAMPs), pathogen-mediated infections, and cellular stress, among other factors [15]. In addition, this member of the CXC chemokine superfamily is characterized by its participation in different stages of cell recruitment during the inflammatory response [16]. Structurally, it presents an ELR/DLR motif that is widely conserved in both higher and lower vertebrates, which allows high affinity binding to IL-8 receptors (IL-8Rs), which are distributed in different fish immune cells [17,18,19].

IL-8Rs are receptors with seven transmembrane domains showing an extracellular N-terminal and intracellular C-terminal. They also have an amino acid motif in the second intracellular loop, called DRY (abbreviation of the three amino acids that compose it), involved in the activation of the G-protein-linked signalling pathway (GPCR) [20,21,22]. In rainbow trout, two kinds of IL-8Rs, namely CXCR1 and CXCR2, have been described through which IL-8 can signal. Both receptors have been found to be differentially expressed in tissues, such as thymus, spleen, gill, and head kidney, in response to PAMPs, pathogens, or cytokines [21]. However, CXCR2 has been found to be highly expressed in muscle rather than in immune relevant tissues, so it may have a potential role in the fish musculo-skeletal system [21]. In rainbow trout smolt, the expression of CXCR1 has been described to increase at the transcript level in response to *Y. ruckeri* [21]. However, its expression at the protein level has not been described in salmonids, and it is not known if this receptor is expressed in early stages of teleost fish development.

The immunomodulation of larval fish has been suggested as a potential strategy for enhancing larval survival by boosting their innate immune responses until their adaptive immune systems are sufficiently developed to effectively combat pathogens [23]. However, the use of biomarkers to assess its effectiveness is essential. In this context, we propose IL-8 and its receptor as potential immunological markers for the early developmental stages of trout. Therefore, in this work, an omCXCR1-derived epitope peptide was designed and characterized, which was used to produce an antiserum that allowed immunodetecting this receptor in rainbow trout larvae. In addition, the antiserum was used to localize the receptor and its ligand (omIL-8) in tissues of 19 dph trout larvae challenged with bacterial LPS as immunostimulant.

## 2. Materials and Methods

### 2.1. In Silico Analysis, Synthesis, Characterization, and Antisera Production

The CXCR1 protein sequence of *Oncorhynchus mykiss* (omCXCR1) was obtained from the UniProt database http://www.uniprot.org/ (accessed on 1 October 2022) corresponding to GenBank access number Q90ZZ2. The methodology used to define the best antigenic peptide was described previously [24]. The candidate epitope was localized in silico using Protter webinterface https://wlab.ethz.ch/protter/start/ (accessed on 15 October 2022) [25] and the 3D model was obtained from RCSB protein data bank https://www.rcsb.org/ (accessed on 19 November 2022). Identification of the antigenic peptide in the model and visualization were carried out using PYMOL (PyMOL Molecular Graphics System, Version 2.0 Schrödinger, LLC., New York, NY, USA). The peptide consisting of 17 residues (NFDTLSCAAQPLSPGAV, corresponding to the region between amino acids 32–48 of omCXCR1 N-terminal), was chosen as candidate for subsequent chemical synthesis as previously described by [26]. As a negative control, a scrambled peptide was synthesized using the same amino acids as omCXCR1 but in a random order (LSVLTCSQPAAADPNGF). The purity and molecular mass of the peptides were confirmed by RP-HPLC and ESI-MS spectrometry, respectively. Polyclonal antibodies were generated against omCXCR1-derived synthetic peptide (omCXCR1*sp*) in six-week-old female CF-1 mice, as previously described [26].

ClustalW alignment was performed with *O. mykiss* CXCR1 (GenBank access number Q90ZZ2) and CXCR2 (GenBank Accession number CDK69051.1) amino acid sequences in order to identify the conserved regions.

### 2.2. Obtaining Trout Larvae and Challenge with LPS

Trout larvae aged 8 days post-hatch (dph), with an average weight of 54 ± 0.01 mg, were obtained from the Río Blanco Fish Farming facilities in Los Andes, Chile. The larvae were transferred to 6 L ponds, with 60 larvae in each pond, and maintained at 10 ± 0.5 °C with oxygen saturation of 90 ± 5%. The larvae were kept under natural photoperiod conditions and fed twice daily with a commercial diet.

The 10, 13, 16, 19, and 33 dph trout larvae were separated into two groups: one group was challenged by immersion in a 1 µg/mL solution of enriched LPS extract from *P. aeruginosa* (donated by A. Dinamarca, Universidad de Valparaíso, Chile) according to previous work [13], while the other group was immersed in freshwater alone as a control. Samples were taken at 2 and 8 h after stimulation, with six larvae from each group being sacrificed by immersion in a 25 mg/L benzocaine (BZ-20, Veterquímica) solution until no signs of movement were observed.

### 2.3. Immunodetection of omCXCR1 in Trout Larvae

To demonstrate that the antiserum could detect the omCXCR1*sp*, dot blot and calibration curve through indirect ELISA were performed as previously described [13,26]. Briefly, for dot blot, the synthetic peptide was seeded on a 0.45 µm nitrocellulose membrane (Thermo Fisher Scientific, Waltham, MA, USA) in amounts of 4, 2, 1, 0.5, 0.25, and 0.125 µg. The membrane was then incubated with the mouse antiserum anti-omCXCR1*sp* at a 1:1000 dilution for 1 h at 20 °C. Incubation with Horseradish Peroxidase (HRP)-coupled mouse anti-IgG (H + L) (Thermo Fisher Scientific), was performed at a 1:7000 dilution for 1 h at 20 °C. Finally, the membrane was revealed by chemiluminescence using the Westar supernova kit (Cyanagen, Bologna, Italy). For the case of the ELISA, starting from 3 ng/µL of the synthetic peptide, serial dilutions were performed in PBS (phosphate-buffered saline) 1×. The antiserum anti-omCXCR1*sp* was used at a 1:2000 dilution for 1 h at 37 °C. The same dilution and conditions were used for secondary antibody in dot blot.

To assess the protein expression levels of IL-8 and omCXCR1 at the time of challenge, an indirect ELISA was performed as above with some modifications. Briefly, 5 ng/µL of total protein of whole larva samples (10, 13, 16, 19, and 33 dph) were seeded on Nunc MaxiSorp™ high protein-binding capacity 96-well ELISA plates. The mouse antiserum anti-omCXCR1*sp* was used at a 1:2000 dilution for 1 h at 20 °C. The same dilution and conditions for secondary antibody mentioned above were used. For the measurement of IL-8, a previously standardized rabbit antiserum anti-omIL-8 was used at a 1:2000 dilution for 1 h at 37 °C [13].

### 2.4. Immunohistochemistry and Immunofluorescence

To localize the omCXCR1 in 19 dph trout larvae’s tissues, immunohistochemistry analyses were performed as previously described [27]. Slides were incubated for 1 h in a wet chamber with the mouse antiserum anti-omCXCR1*sp* (at 1:50 dilution). After washing, samples were incubated with a secondary anti-mouse biotinylated antibody (DAKO) at a 1:100 dilution for 1 h and then for 1 h with a peroxidase–avidin–biotin complex (DAKO). Contrast staining was performed using Hansen haematoxylin solution. Finally, the samples were dehydrated with an ascending gradient ethanol battery and mounted with Neomount (Merck, Rahway, NJ, USA). Slides were analysed using the microscope Eclipse Ni-E (Nikon Corp., Tokyo, Japan). Stain separation for DAB (3,3-diamino benzidine) (Sigma-Aldrich, St. Louis, MO, USA) and haematoxylin and stained area percentage estimation were carried out using the Color Deconvolution plugin and Threshold tool, respectively, in ImageJ software, version 1.54f (NIH, Bethesda, MD, USA).

In order to colocalize the presence of the omCXCR1 with IL-8 in skin, gills, and gut tissue of 19 dph trout larvae, indirect immunofluorescence was performed as previously described [13]. Slides were first incubated for 6 h with the rabbit antiserum anti-IL-8 (at 1:500 dilution). After washing with PBS 1×, slides were incubated for 6 h with mouse antiserum anti-omCXCR1*sp* (at 1:500 dilution). Then, slides were incubated with Goat Anti-Rabbit IgG (H + L) Cross-Adsorbed Secondary Antibody (Invitrogen, Waltham, MA, USA) and Alexa Fluor 635 and Goat anti-Mouse IgG (H + L) Cross-Adsorbed Secondary Antibody, Alexa Fluor 568 (Invitrogen), both diluted 1:400. SYTO 9 fluorophore (Invitrogen) was used as a nucleus stain. The tissues were analysed using a Leica TCS SP5 II Spectral Confocal Microscope (Leica Microsystems Inc., Wetzlar, Germany). The colocalization analysis was performed using the Coloc module found within the Imaris 7.4.2 software suite (Bitplane Scientific Software, Belfast, UK), which utilizes a threshold model for analysis. Pearson’s correlation coefficient was calculated for the specified regions of interest.

### 2.5. Statistical Analysis

Statistical analysis was conducted using GraphPad Prism 8.0. A one-way ANOVA was used to compare the expression levels of omIL-8 and omCXCR1 with respect to the control group and corresponding time points post-LPS challenge in rainbow trout larvae across different days of treatment. Pearson’s correlation test was used in rainbow trout larvae tissues in the immunofluorescence images to analyse the association between IL-8 and omCXCR1-positive cells.

Differences were considered significant when *p* < 0.01 (****) or *p* < 0.05 (**).

### 2.6. Ethics Statement

Fish were maintained and handled following the guidelines of experimental procedures approved by the Ethics Committee of the Pontificia Universidad Católica de Valparaíso (Permit Number BIOEPUCV-BA 280–2019).

## 3. Results

### 3.1. Characterization of CXCR1 of Rainbow Trout and Selection of Epitope Peptide

**Figure 1:**
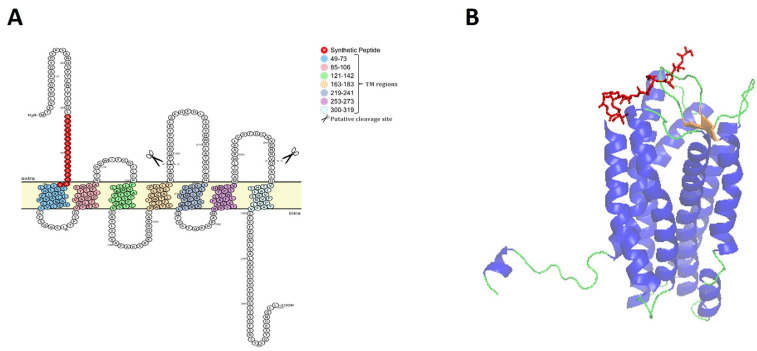
Structure and identification of epitope peptide from omCXCR1. (**A**) The predicted topology and sequence of omCXCR1 is shown with putative seven transmembrane segments positioned in the cell membrane. The amino acid sequence of the chosen epitope peptide is indicated in red. Furthermore, two conserved sites for cathepsin G and elastase in extracellular regions are indicated. (**B**) Three-dimensional structure of omCXCR1, where the chosen epitope peptide is shown in red.

The IL-8 receptor or CXCR1 of rainbow trout (omCXCR1) is a polypeptide of 359 amino acid residues (available in GenBank accession number: NP_001117751.1) that is composed of seven putative transmembrane segments and three extracellular loops (Figure 1A), which is a highly conserved structure for teleost chemokine receptors. In addition, an in silico analysis with putative proteolytic cleavage of omCXCR1 revealed two conserved sites for cathepsin G (^184^NDAF…TPQ_190_) and elastase (^291^RNRV…DLA^297^) in two extracellular regions (Figure 1A).

Since there is no 3D model described for omCXCR1 of teleost species, the crystal structure of human CXCR1 obtained by X-ray diffraction (SMTL ID: 6lfo1) was used for visualizing accessible antigenic peptides. The putative 3D structure of omCXCR1 is shown in Figure 1B. The model shows the presence of eight alpha-helix structures.

Analysis of hydrophobicity, accessibility, and chain flexibility properties were applied to identify the best antigenic peptide of omCXCR1 (Supplementary Figure S1). The chosen peptide was localized in the N-terminal region that is facing the extracellular space (Figure 1).

The chosen peptide epitope of omCXCR1 used to generate polyclonal antibodies in mice showed a low identity with the amino acid sequence of omCXCR2 (Supplementary Figure S2).

### 3.2. Characterization of CXCR1 Mice Antisera

**Figure 2:**
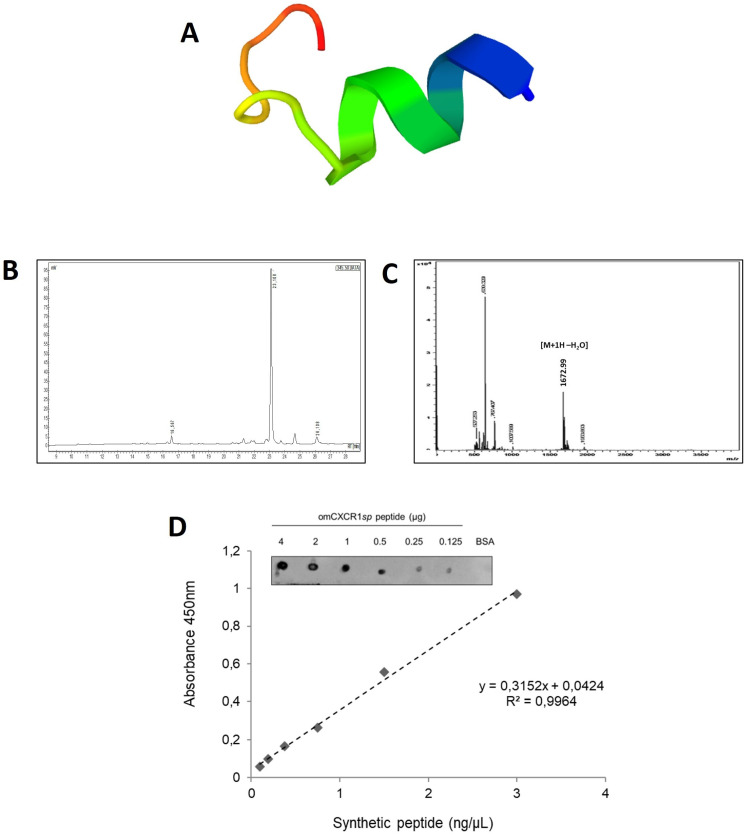
Characterization of synthetic peptide epitope antisera to detect omCXCR1. (**A**) Three-dimensional structure of epitope peptide of omCXCR1. (**B**) Spectra of reverse-phase high-performance chromatography (RP-HPLC) revealed a purity of the epitope peptide higher than 90%. (**C**) ESI-MS showing the molecular ion [M+1H-H_2_O] with *m*/*z* of 1672.99 Da. (**D**) Mouse antiserum anti-omCXCR1*sp* was characterized by dot blot and indirect ELISA, performing serial dilutions of synthetic peptides from 4 to 0.125 µg/µL and from 3 to 0.09 ng/µL, respectively.

The chemically synthesized epitope peptide of omCXCR1 presented a short α-helical structure (Figure 2A). This peptide was purified by RP-HPLC to a purity higher than 90% and presents a molecular mass of 1672.99 Da (Figure 2B,C). Then, the synthetic peptide was used as an epitope vaccine for the development of omCXCR1 antiserum in mice. After the immunization of mice, the antiserum obtained displayed strong immunoreactivity against the synthetic peptide derived from omCXCR1 (at 4 to 0.125 μg) and no immunoreactivity to bovine serum albumin (BSA) was observed (Figure 2D). The recognition of omCXCR1*sp* was confirmed by ELISA, showing a linear regression with an R^2^ = 0.9964.

The scramble peptide was purified by RP-HPLC to a purity higher than 90% and presents a molecular mass of 1690 Da (Supplementary Figure S3). The mouse antiserum showed no cross-reaction with the scramble peptide. The preimmune serum showed negligible immunoreactivity against omCXCR1*sp*.

### 3.3. Effect of LPS Challengue in the Protein Expression and Location of omCXCR1 and omIL-8 in Trout Larvae

LPS was used as an immunostimulant model to induce the immune response of rainbow trout larvae of 10, 13, 16, 19, and 33 dph by immersion. This effect was evaluated through the measurement of omIL-8 and omCXCR1 protein expression using a previously validated omIL-8 rabbit antisera [13] and the omCXCR1sp antisera characterized in this study.

**Figure 3:**
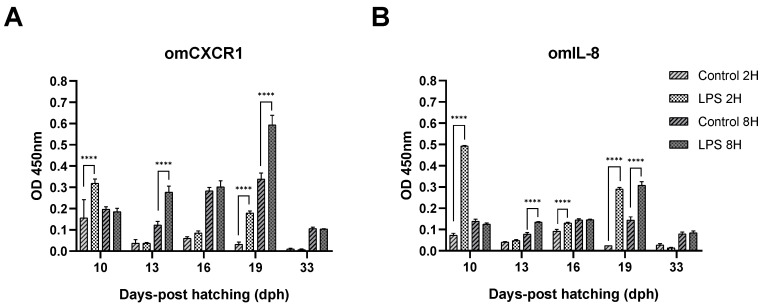
Effect of LPS challenge on the protein expression of omIL-8 and omCXCR1 in rainbow trout larvae at 10, 13, 16, 19, and 33 days post-hatching (dph). Indirect ELISA showing omCXCR1 (**A**) and omIL-8 (**B**) bioavailability in trout larvae 2 h and 8 h after LPS challenge. Statistically significant differences are indicated with **** *p* < 0.01.

From the results obtained, it can be noted that 1 µg/mL of LPS was able to significantly stimulate the omCXCR1 protein expression in trout larvae at 10 and 19 dph after 2 h and at 13 dph 8 h after challenge. In the case of omIL-8 protein expression, it was significantly stimulated in trout larvae at 10, 16, and 19 dph after 2 h and in trout larvae 13 and 19 dph 8 h after challenge. In addition, the more pronounced effect was observed in larvae at 19 dph, where the immunestimulation was reflected by a significant increase in omIL-8 and omCXCR1 levels (15 and 6 times higher than the control at 2 h, respectively) (Figure 3A,B). At 8 h, a significant twofold increase in omIL-8 and omCXCR1 levels was observed with respect to the control (Figure 3A,B).

**Figure 4:**
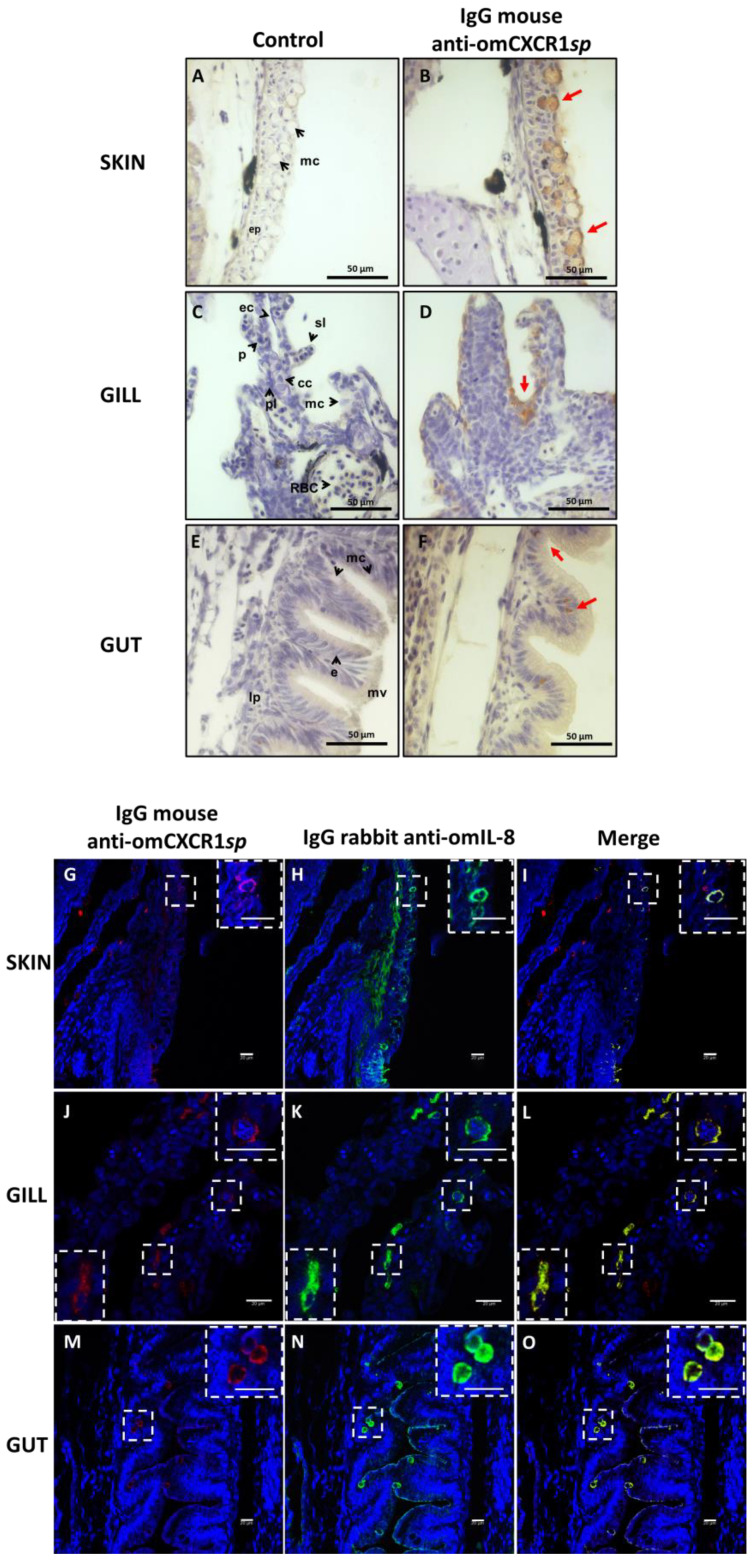
Location of omIL-8 and omCXCR1 in rainbow trout fry at 19 days post-hatching after LPS challenge. Immunolocalization of omCXCR1/IL-8 in tissues of rainbow trout larvae 8 h after LPS challenged. Above: Representative photos of trout larvae at ×40 magnification illustrating the localization by immunohistochemistry (**A**–**F**) of omCXCR1 in fish skin, gill, and gut in control (**A**,**C**,**E**) and LPS-challenged larvae (**B**,**D**,**F**). Red arrows denote corresponding cells which are omCXCR1 positive. The letters in black are defined as follows: mc: mucous cells; ep: epidermis; ec: epithelial cells; p: pillar cell; sl: secondary lamellae; pl: primary lamellae; cc: chloride cells; RBC: red blood cells; e: enterocyte; mv: microvilli, and lp: lamina propia. Bar: 50 μm. Below: Co-localization of omCXCR1 with omIL-8 is shown in fish skin (**G**–**I**), gill (**J**–**L**), and gut (**M**–**O**). omCXCR1 was detected using mouse antiserum anti-omCXCR1*sp* (1:500 dilution) and Alexa Fluor 568 (**G**,**J**,**M**), and omIL-8 was detected using rabbit antiserum anti-omIL-8 (1:500 dilution) and Alexa Fluor 635 (**H**,**K**,**N**). Nuclear staining was performed using SYTO 9. Bar: 20 μm.

Subsequently, the localization of omIL-8 and omCXCR1 in the skin, gills, and intestine of 19 dph rainbow trout larvae 8 h after challenge was studied using immunohistochemistry and immunofluorescence (Figure 4). Cells present in the epidermis of the skin showed positive staining for omCXCR1. This staining is particularly evident in mucosal cells, such as goblet cells. In gills of trout larvae, positive epithelial cells (probably pavement cells) were observed on the gill lamellae. Additionally, pillar cells also showed positive staining. In the gut, goblet cells and enterocytes exhibited positive staining.

In all mucosal tissues, the colocalization of the omCXCR1 receptor with omIL-8 was observed by immunofluorescence (Figure 4 below). Pearson’s correlation was used to assess the colocalization, indicating a higher colocalization of the omCXCR1 receptor and omIL-8 in gut tissue compared to skin and gills (Supplementary Figure S4). This colocalization was mainly observed in mucosal cells of the skin and gut followed by epithelial and endothelial cells modified as pillar cells in the gills.

## 4. Discussion

Salmonids in early developmental stages are known to be highly vulnerable to different pathogens such as fungi, bacteria, and viruses [28,29]. During this time, fish larvae rely solely on their innate immune system as their adaptive immune system is still developing, making them highly susceptible to these infectious diseases. Therefore, the use of common immunomodulators molecules could be a strategy for strengthen the innate immune response of salmonid larvae. However, the influence of these compounds during early development steps requires the evaluation of larval immunocompetence.

In the current study, the use of omIL-8 chemokine and its receptor omCXCR1 allowed us to verify that the administration of *P. aeruginosa* LPS increased larval immune response. This result is consistent with findings from an experiment with zebrafish (*Danio rerio*) larvae, where larvae of 2, 5, and 10 days post-fertilization (dpf) treated with *Escherichia coli* or *P. aeruginosa* LPS showed increase in both survival rates and expression of IL-1β pro-inflammatory cytokine [30]. However, the origin of the LPS greatly influences the type of immunostimulation expected. In the study by Novoa et al. [30], stimulation with LPS from *P. aeruginosa* showed better results than LPS from *E. coli*. Similarly, Santos et al. [31] reported that treatment of *D. rerio* larvae with LPS from *Photobacterium damselae* did not cause mortality, whereas LPS from *Tenacibaculum maritimum* resulted in a mortality rate of 80.6% in zebrafish larvae [31]. Consequently, certain types of LPS may trigger excessive inflammatory responses, while others can maintain their adjuvant effect for adaptive immunity [32]. Therefore, the source of LPS significantly impacts the type and effectiveness of immunostimulation. Future research is needed to determine whether different types of LPS can offer enhanced immunostimulant effects in freshwater salmon hatcheries because improved immunostimulation may lead to increased resistance to infections, higher growth rates, and overall improved health and survival rates of salmon in hatcheries.

LPS is the major constituent of the outer layer of Gram-negative bacteria. This modulator enhances the production of chemokines and proinflammatory cytokines, which activate the recruitment and differentiation of immune cells and trigger antibacterial mechanisms, including complement, extracellular proteases, and reactive oxygen species (ROS) [33,34]. Whether this holds true for fish remains unclear as the LPS recognition mechanism in fish is noticeably different than in mammals and not yet well understood [31,35]. Fish are resistant to the toxic effects of high doses of LPS because they respond to LPS via a Tlr4/Myd88-independent pathway, leading to increased levels of pro-inflammatory cytokines, such as IL-8 and its cognate receptor [36]. Additionally, some authors suggest that crude LPS preparations stimulate pro-inflammatory cytokines in rainbow trout macrophages not primarily through endotoxin but rather through the presence of peptidoglycans [37].

The route of immunostimulants compounds administration is another factor that must be carefully considered to understand the effects on the immune system of fish during early developmental stages. Unlike adult fish, which can receive immunostimulant molecules by injection or dietary incorporation, the application of these compounds during the larval stage is limited to immersion or bathing [38,39,40]. To support the immunostimulation strategy in salmonid larvae, our results demonstrated an increased expression of omIL-8 and its cognate receptor mainly at 19 dph in mucosal tissues, which are primarily responsible for recognizing PAMPs in the aquatic environment. Previous studies on olive flounder fry have shown that IL-8 expression increases significantly only after 15 dph, primarily in the gills. This mucosal tissue is notably faster in the immunological response to infection with viral haemorrhagic septicemia virus than spleen or kidney [41].

In all mucosal tissues of trout larvae (skin, gill, and gut), the immunodetection of omCXCR1 and omIL-8 was primarily observed in mucous cells, such as goblet, epithelial, and endothelial cells modified as pillar cells in LPS-challenged larvae. Similar studies conducted by Oehlers et al. [42] during the development of zebrafish revealed that IL-8 and its receptors (CXCR1 and CXCR2) are expressed in leukocytes and epithelial cells of the zebrafish intestine. This expression was increased under inflammatory conditions caused by chemical or bacterial challenge. Additionally, previous studies have described the expression of IL-8 in mucous cells of rainbow trout larvae [43].

Furthermore, the colocalization reactivity analysis indicated a higher colocalization of omCXCR1 merged with omIL-8 in gut tissue compared to skin and gills. These results support the idea that the main immunocompetent tissues during the larval stage are the mucosal tissues. Early lymphocyte colonization of mucosal immune organs in rainbow trout (*O.s mykiss*) is readily recognized after a few dph, such as in the gut wall after 13 dph [43,44]. Furthermore, previous studies on the ontogeny of the adaptive immune system in rainbow trout have shown that the intestine and gills are among the earliest organs to express markers associated with this type of immunity, such as MHCII or IgT [43]. Therefore, we propose that these tissues should be the primary targets for studying immunostimulants during these early development stages.

Samanta et al., [45] described for the first time that IL-8 was able to regulate its receptor through the involvement of lysosomal enzymes. This mechanism is related to avoiding IL-8 overstimulation during inflammatory processes and, therefore, could be highly relevant in the control of chemotactic responses induced by IL-8 [45]. Later, Hartl et al. [46] described the serine proteases that are involved in the cleavage of surface CXCR1 in human neutrophils. Interestingly, different serine proteases that cause proteolytic cleavage of CXCR1, such as elastase and cathepsin G, were identified during in silico analysis to find putative proteolytic cleavage of omCXCR1 [46]. However, future studies are required to confirm if this post-translation modification is performed in omCXCR1.

## 5. Conclusions

The findings of this work underscore the efficacy of LPS immersion as a method to activate the innate immune system in trout larvae. Furthermore, we propose omIL-8 and its receptor as molecular markers for evaluating immunostimulation in the early developmental stages of salmonids. Notably, it identified that 19 dph is a critical period for the development of robust responses to LPS stimulation, particularly in mucosal tissues. This advancement is pivotal in devising effective strategies for infectious disease management and prevention in freshwater salmon hatcheries.

## Data Availability

Dataset available on request from the authors.

## Supplementary Materials

The following supporting information can be downloaded at: https://www.mdpi.com/article/10.3390/ani14192878/s1, Figure S1: Hydrophobicity, accessibility, and flexibility profiles for omCXCR1 protein. Figure S2: Alignment of *Oncorhynchus mykiss* CXCR1 and CXCR2 amino acid sequences. Figure S3: Colocalization analysis of fluorescent probes in selected tissues. Figure S4: Colocalization analysis of fluorescent probes in selected tissues.
