# Supplementary material for: Detection and Localization of IL-8 and CXCR1 in Rainbow Trout Larvae in Response to Pseudomonas aeruginosa Lipopolysaccharide
Source: Animals (Basel). 2024 Oct 6;14(19):2878. doi: 10.3390/ani14192878 (PMC11475925; doi:10.3390/ani14192878)
Supplement: Supplementary file 1 [file animals-14-02878-s001.zip › animals-3183671-supplementary.pdf]

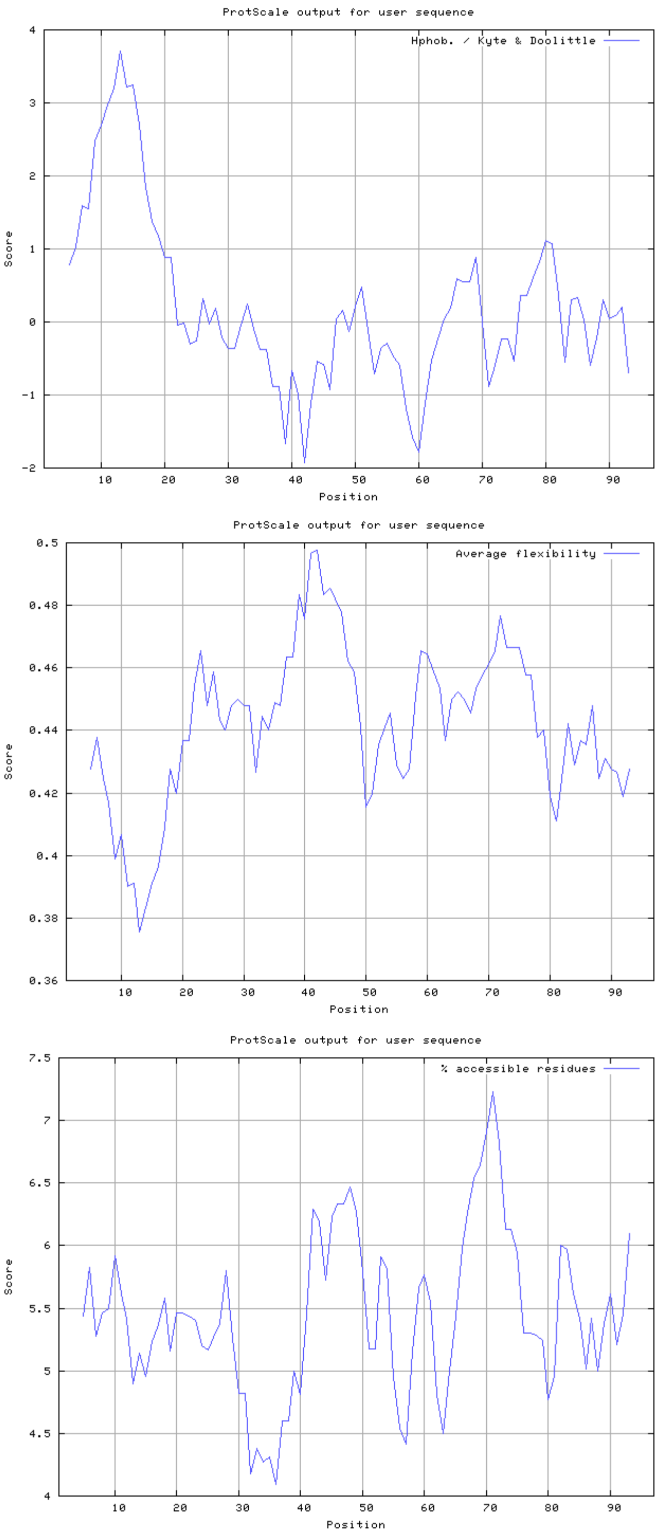

Supplementary figure S1. Hydrophobicity, accessibility and flexibility profiles for omCXCR1 protein.

```
CXCR1   MTEVLDYDYKADYDYKSANDSYYFNITSFDL----NFDTLSCAAQPLSPGAVIFLCVLHV    56
CXCR2   -MEMQEI------DY---NDALYSDIFNFTYPPIDELKAAPCSVSILGLSS-VGLMVTYI    49
          *: :       **   **: * :* .*      ::.:  *:.. *. .: : * * ::

CXCR1   AIFLLAVPGNLLVGLVIGFSQQSLTPSDVYLFHLTVADGLLALTLPFWAANTLHGWIFGD    116
CXCR2   IVFVLSVLGNSVVIYVMCCLARSRTTTDIYLMHLAMADLLFSLTLPFWAVYVYSHWIFGT    109
         :*:*:* ** :*  *:    :* * :*:**:**::** *::*******. .   ****

CXCR1   FLCKFLSLVMEASFYTSILFLVCISVDRYLVIVRPAKSRKGHRRACRWYACTFIWTLGGA    176
CXCR2   FLCKLLSGLQDASFYSGVFLLACISVDRYLAIVKTTQALTQ-RRHLVGKVCGAVWLGAGL    168
        ****:** : :****:.:::*.********.**: ::: .  **     .*  :*  .*

CXCR1   LSLPALFNDAFT--PQRGGPTRCAEHFDLSSATHWRLATRGLRHILGFLLPLVIMVACYS    234
CXCR2   LSLPVVLQREAIQLEDLSDQTICYENLTASSSNQWLVFVRVLRHTLGFFLPLAVMVVCYS    228
        ****.:::       : .. * * *::  **:.:* : .* *** ***:***.:**.***

CXCR1   ITVARLLQT--HGFQKHRAMRVIIAVVFAFLLCWTPLHMTVMADTLMRAKLVR-FDCAVR    291
CXCR2   CTATTMFRGMRNADHKHKAMRVILAVVLAFVLCWLPCNVSVLVDTLMRGGLLGEETCEFR    288
         *.: :::   :. :**:*****:***:**:*** * :::*:.*****. *:    * .*

CXCR1   NRVDLALQVTHSLALVHSFVNPVLYAFVGEKFRGNLGALVRKSRGPERGSSSGFSRSTS-    350
CXCR2   NSVSVALYVTKGIAFTHCAVNPVLYAFIGQKFRNQLLLMLHKHGLISKRVLAAYRRGSAP    348
        * *.:** **:.:*:.*. ********:*:***.:*  :::*    .:   :.: *.::

CXCR1   ---QTSEGNGLL--      359
CXCR2   STVSQRSRNTFISL      362
           .  . * ::
```

Supplementary figure S2. Alignment of *Oncorhynchus mykiss* CXCR1 and CXCR2 amino acids sequences. The alignment of omCXCR1 (GenBank accession number Q90ZZ2)and omCXCR2 (GenBank accession number CDK69051.1)amino acid sequences was performed using ClustalW. Conserved amino acids are shown with asterisk (*). The peptide epitope (omCXCR1*sp*) is shown in an orange box.

A

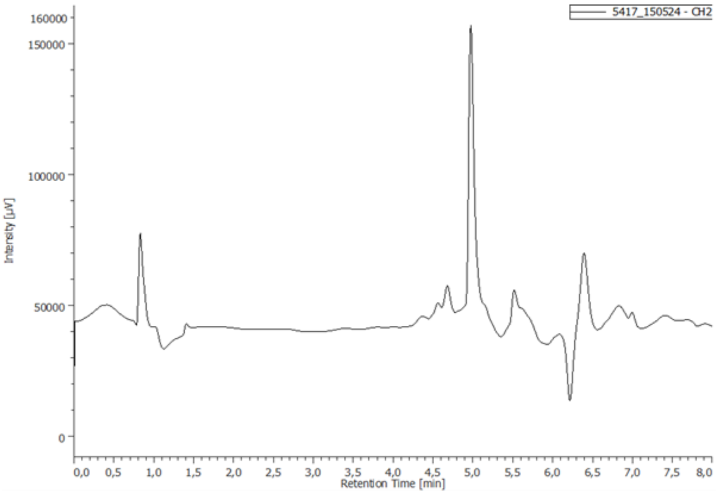

B

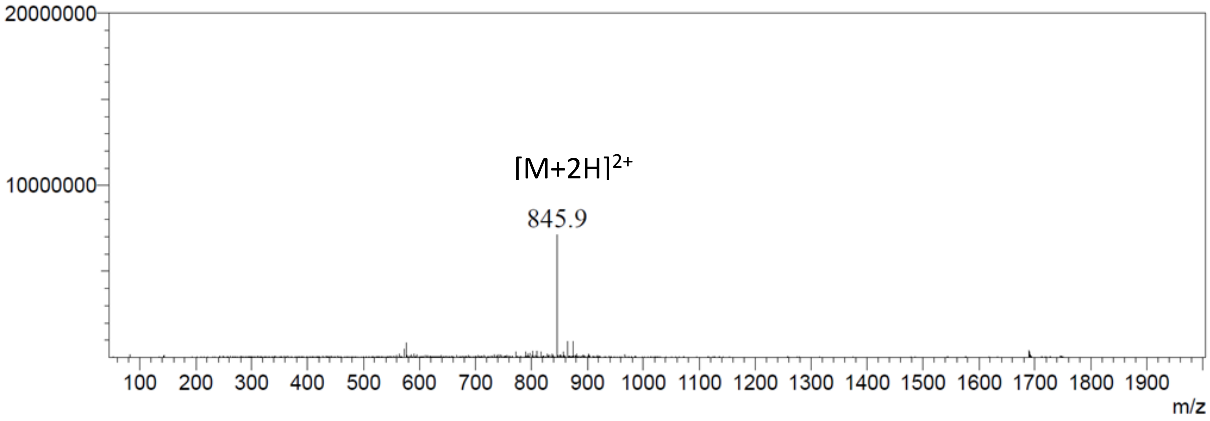

Supplementary figure S3. Characterization of synthetic scramble peptide. A) reversed-phase HPLC (RP-HPLC). B) mass spectrometry spectrum (ESI-MS) of synthetic peptide showing the molecular ion $[M+2]^{2+}$.

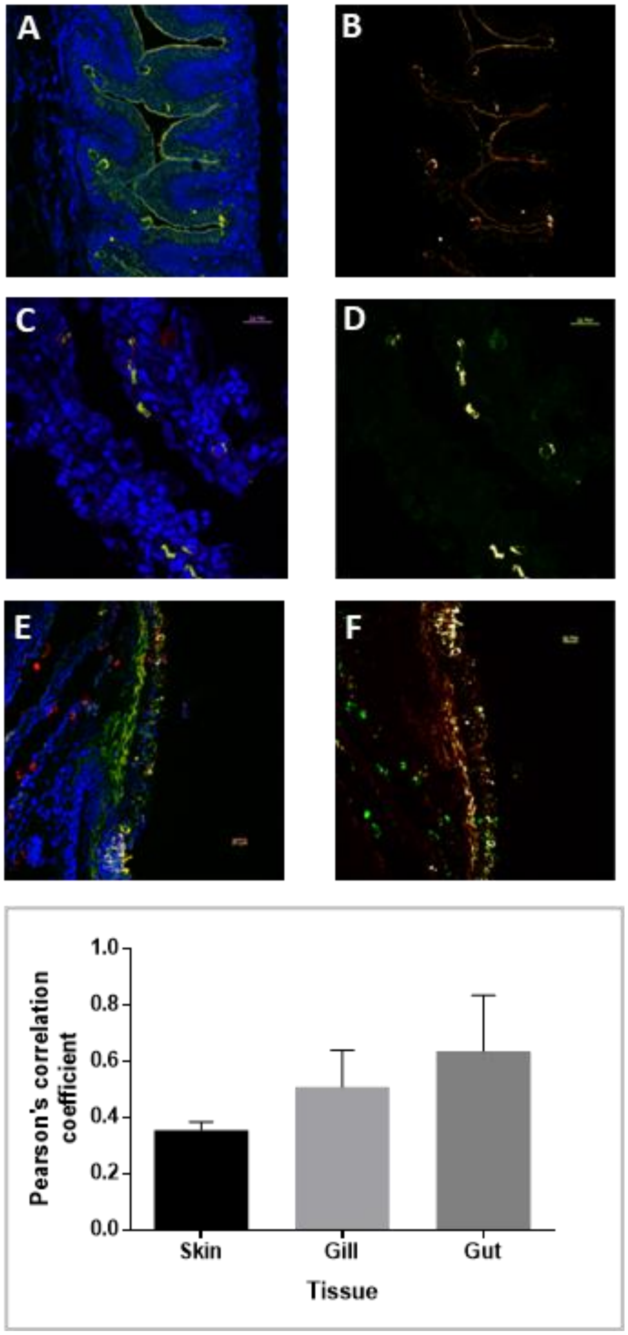

Supplementary figure S4. Colocalization analysis of fluorescent probes in selected tissues. Pearson's correlation was used to assess the colocalization of two specific fluorescent probes, omCXCR1 (Alexa fluor 568) and omIL-8 (Alexa fluor 635), within regions of interest in fish larvae skin, gill, and gut samples. *Bar* 20µm.
